# Sleep in Lennox–Gastaut Syndrome: A Scoping Review

**DOI:** 10.3390/children12121676

**Published:** 2025-12-10

**Authors:** Debopam Samanta

**Affiliations:** Division of Child Neurology, Department of Pediatrics, University of Arkansas for Medical Sciences, Little Rock, AR 72202, USA; dsamanta@uams.edu

**Keywords:** Lennox–Gastaut syndrome, sleep architecture, cyclic alternating pattern, sleep-disordered breathing, circadian rhythm, developmental epileptic encephalopathy

## Abstract

**Background and Objective**: Lennox–Gastaut syndrome (LGS) is a severe developmental and epileptic encephalopathy characterized by multiple seizure types, distinctive electroencephalography (EEG) abnormalities, and cognitive impairment. Sleep disturbances are highly prevalent in LGS and contribute substantially to reduced quality of life. However, no comprehensive analysis has yet been conducted to systematically examine key aspects of sleep—including architecture, microstructure, sleep-disordered breathing, and circadian regulation—leaving critical knowledge gaps. To address this, we conducted a scoping review to map the current evidence on sleep abnormalities in LGS and to identify priorities for future research. **Method**: A scoping review was conducted following PRISMA-ScR guidelines. PubMed, Embase, Ovid, and ClinicalTrials.gov from inception to October 2025 for studies evaluating sleep parameters in individuals with LGS or mixed epilepsy cohorts with ≥50% LGS cases. Eligible designs included observational and interventional studies using polysomnography, video-EEG, actigraphy, or sleep questionnaires. Data were synthesized narratively due to heterogeneity, and methodological quality was assessed using relevant Joanna Briggs Institute (JBI) checklists. **Results**: After screening 1242 articles, eleven studies met inclusion criteria, spanning 1986–2025 and conducted across four continents. Most were small single-center observational studies (5–16 LGS participants) using polysomnography as the primary assessment, with others employing wearable monitoring, surface and intracranial EEG, or circadian biomarker analyses. Across studies, individuals with LGS demonstrated markedly disrupted sleep architecture—notably reduced or absent rapid eye movement (REM) sleep, fragmented non-rapid eye movement (NREM) sleep, and attenuated spindles. Microstructural analysis showed elevated cyclic alternating pattern (CAP) rates, with epileptiform discharges clustering in CAP phase A. Sleep-disordered breathing (SDB) was common, particularly in adults, and associated with tonic seizures and central apneas. Circadian rhythm dysregulation, including altered melatonin and cortisol profiles, was also reported. A feasibility study demonstrated that home-based wearable devices and sleep apnea monitors were both acceptable and practical for use in children with LGS. No interventional studies have evaluated whether addressing sleep abnormalities modifies seizure or cognitive outcomes. **Interpretation**: Sleep in LGS is profoundly disrupted at both macrostructural and microstructural levels. These abnormalities may exacerbate seizure burden, cognitive impairment, and SUDEP risk, representing a potentially modifiable contributor to disease severity. Larger, prospective studies integrating polysomnography, wearable monitoring, and interventional approaches are needed to clarify causal mechanisms and therapeutic potential.

## 1. Introduction

Lennox–Gastaut syndrome (LGS) is a severe developmental and epileptic encephalopathy (DEE) characterized by multiple seizure types, interictal electroencephalography (EEG) patterns of diffuse slow spike–wave and generalized paroxysmal fast activity, and typically significant cognitive impairment [1]. It usually presents between 1 and 8 years of age and may evolve from other DEEs [2].The etiologies of LGS are heterogeneous—spanning structural, genetic, metabolic, and unknown causes—but converge on widespread network dysfunction leading to drug-resistant epilepsy (DRE) and profound neurodevelopmental impairment [3]. While intractable seizures define the syndrome, comorbid sleep disturbances are highly prevalent and contribute substantially to reduced health-related quality of life for both patients and caregivers [4]. Sleep and epilepsy may interact bidirectionally in LGS, whereby nocturnal seizures fragment sleep and poor or unstable sleep may, in turn, aggravate seizure frequency and related cognitive–behavioral difficulties [5]. Moreover, uncontrolled nocturnal seizures increase the risk of sudden unexpected death in epilepsy (SUDEP) [6]. Sleep disruption also extends to caregivers—up to 90% report inadequate or disturbed sleep—amplifying fatigue, mood symptoms, attentional difficulties, and overall family burden [4,7].

Despite recognition of the link between sleep and seizures in LGS for more than three decades, the literature remains sparse, fragmented, and largely descriptive. No prior review has comprehensively synthesized available evidence on how different aspects of sleep—macrostructure, microstructure, sleep-disordered breathing, and circadian regulation—interact with seizure burden, neurocognitive outcomes, and overall disease trajectory in LGS.

To address this gap, we conducted a scoping review to systematically map the existing evidence on sleep in LGS across all age groups. The objectives were to (1) summarize current knowledge regarding sleep architecture, microstructure, and comorbid sleep disorders; and (2) identify gaps and priorities for future research, including opportunities to develop sleep-based biomarkers and therapeutic targets in this complex DEE.

## 2. Methods

The scoping review was conducted in accordance with PRISMA-ScR guidelines and was not registered.

### 2.1. Eligibility Criteria

Studies were included if they enrolled individuals with a diagnosis of LGS, regardless of age or etiology, or mixed complex epilepsy cohorts in which at least 50% of participants had LGS. We did not exclude individuals based on the presence of other comorbid conditions. Eligible studies assessed sleep-related parameters, including macrostructural measures (e.g., distribution of rapid eye movement (REM) and non-rapid eye movement (NREM) stages, total sleep time [TST], total time in bed [TIB], sleep efficiency [ratio of TST to TIB], sleep latency [time from lights-out to sleep onset], wake after sleep onset [WASO], and REM latency), microstructural features (e.g., cyclic alternating pattern [CAP]), nocturnal seizure patterns, and interictal epileptiform discharges (IEDs) in relation to sleep cycles, as well as sleep-disordered breathing (SDB) or circadian rhythm alterations.

### 2.2. Sleep Macrostructure and Architecture

Sleep architecture (macrostructure) refers to the organization of NREM (N1–N3) and REM stages across sleep cycles, while sleep microstructure examines finer oscillatory dynamics reflecting brain stability, arousal regulation, and sleep quality [8]. A key microstructural feature of NREM sleep is the Cyclic Alternating Pattern (CAP), which alternates between Phase A (transient activation) and Phase B (stable NREM), recurring every 20–60 s [9]. Phase A is subclassified into A1 (stable slow waves), A2 (mixed slow and fast components), and A3 (desynchronized fast activity), with the CAP rate—the proportion of NREM occupied by CAP—indicating sleep fragmentation. Predominant A1 phases reflect restorative sleep, whereas higher A2/A3 activity indicates disrupted sleep. REM density, measured from electrooculography signals, represents the frequency of rapid eye movements per unit time during REM and reflects REM sleep intensity.

Apneas and hypopneas were scored according to the updated American Academy of Sleep Medicine (AASM) 2012 criteria [10]. In adults, an apnea was defined as a ≥90% drop in airflow from pre-event baseline, measured by an oronasal thermal sensor, positive airway pressure (PAP) device flow, or alternative sensor, lasting ≥10 s. Apneas can be further classified as obstructive, in which airflow ceases due to upper airway obstruction despite ongoing respiratory effort, or central, in which airflow stops due to absent respiratory effort, often related to impaired brainstem control. Hypopneas were defined as a ≥30% drop in airflow for ≥10 s, associated with either ≥3% oxygen desaturation or an arousal. Classification of hypopneas as obstructive or central in adults is optional. In children, apneas were defined as a ≥90% drop in airflow for ≥2 breaths and classified as obstructive, central, or mixed. Central apneas in children required absence of inspiratory effort plus at least one of the following: duration ≥20 s, ≥3% oxygen desaturation or arousal, or (for infants <1 year) a decrease in heart rate below defined thresholds. Hypopneas in children were defined as a ≥30% drop in airflow for ≥2 breaths, associated with ≥3% oxygen desaturation or an arousal. Surrogates of arterial PCO_2_ (end-tidal or transcutaneous) were used when applicable. The apnea–hypopnea index (AHI) quantifies the average number of apneas and hypopneas per hour of sleep and is commonly used to assess the severity of sleep-disordered breathing. Because several included studies were conducted prior to the AASM 2012 [10] update, apnea and hypopnea events were extracted from each study based on the scoring methods reported in the original publications.

Included study designs comprised observational studies (cross-sectional, case–control, or cohort), interventional trials, and case series with ≥3 participants, conducted in clinical, neurophysiological, or interventional settings using polysomnography (PSG), video-EEG, actigraphy, or sleep questionnaires. Exclusion criteria were animal studies, single case reports, reviews, editorials, or studies not reporting sleep-specific outcomes in LGS, as well as those describing only nocturnal tonic seizures captured on EEG or PSG without sleep stage–specific information. Only studies published in English were considered.

### 2.3. Information Sources and Search Strategy

A comprehensive search of PubMed, Embase, Ovid, and ClinicalTrials.gov was conducted from database inception to October 2025. The search combined controlled vocabulary and free-text terms related to LGS and sleep. The primary search string, adapted for each database, was: (“Lennox-Gastaut” OR “Lennox Gastaut” OR “epileptic encephalopathy”) AND (“sleep” OR “polysomnography” OR “REM” OR “NREM” OR “sleep architecture” OR “cyclic alternating pattern” OR “sleep disorder” OR “sleep apnea” OR “circadian” OR “melatonin”). Additional studies were identified through reference lists of included articles and relevant reviews.

### 2.4. Study Selection and Synthesis

All citations were imported into EndNote for duplicate removal and screening. Titles and abstracts were reviewed for relevance, followed by full-text assessment against eligibility criteria, with reasons for exclusion documented at the full-text stage. Due to heterogeneity in study designs and outcome measures, quantitative meta-analysis was not feasible; findings were synthesized narratively and organized thematically across domains. Methodological rigor and reporting quality were assessed using the Joanna Briggs Institute (JBI, Adelaide, South Australia, Australia) checklist for analytical cross-sectional studies, case–control, case series, and cohort studies, as appropriate.

## 3. Results

### 3.1. Study Selection and Characteristics

**Figure 1:**
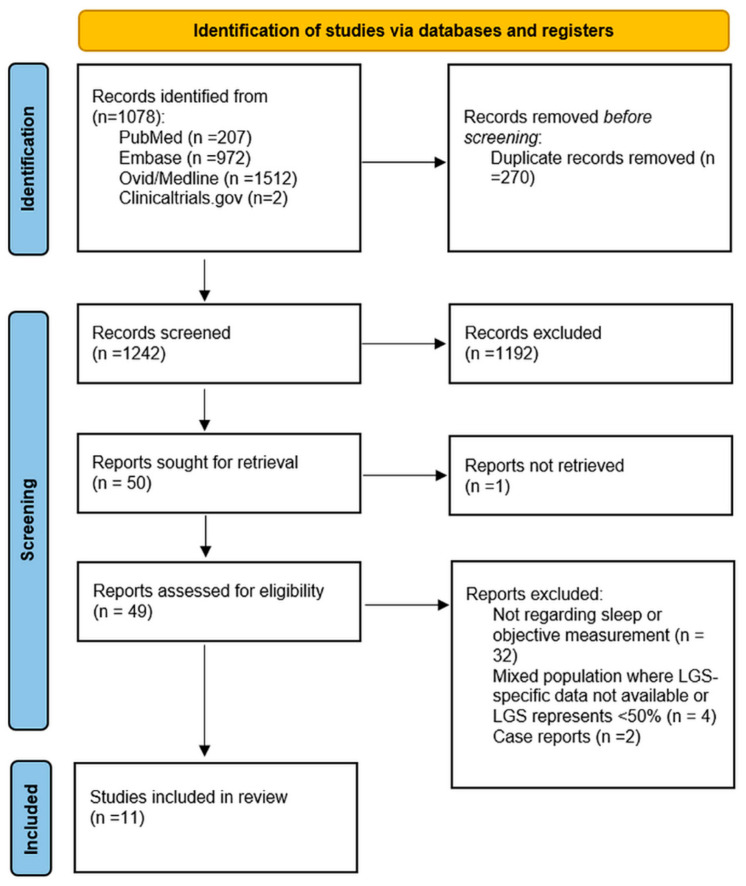
PRISMA flow diagram illustrating the study selection process.

After screening 1242 articles, eleven studies met inclusion criteria, comprising nine full-length publications, one conference abstract, and one registered but unpublished clinical trial (Figure 1). The studies, conducted between 1986 and 2025, were carried out across Europe, Asia, Australia, and North America. (Table 1) Most were single-center observational studies, including six retrospective and three prospective designs. Sample sizes were small, ranging from 5 to 16 patients with LGS, with or without control groups (epilepsy or healthy populations). Two studies included mixed DEE cohorts with more than 50% of participants having LGS. Two studies focused specifically on adults with LGS, while the remaining studies included pediatric or mixed-age cohorts.

Polysomnography (PSG) was the primary assessment method in eight studies, with one study using ambulatory wearable monitoring and another assessing circadian biomarkers (melatonin, cortisol, and body temperature). The most common objectives were characterization of sleep architecture, microstructural correlates of epileptiform discharges, and identification of respiratory or circadian abnormalities.

### 3.2. Sleep Macrostructure and Architecture

Across studies, patients with LGS consistently exhibited marked alterations in sleep architecture compared with healthy or epilepsy controls. In one study, REM sleep was either absent or profoundly reduced in 6 out of 11 children with LGS, with total REM proportion significantly lower than in both epilepsy controls and healthy subjects [11]. These findings were replicated in another study, where REM sleep duration was reduced by more than half compared to controls (55 vs. 116 min), accompanied by a compensatory increase in slow-wave sleep [13]. The total sleep time, wake after sleep onset, and sleep latency did not differ significantly. In contrast, another study reported reductions in time in bed, total sleep time, and sleep efficiency, along with increased REM latency and a higher number of stage shifts per hour [12]. The study by Horita et al. in younger children similarly demonstrated reduced REM percentage and REM density [15]. Sleep spindles were often absent or markedly attenuated in 60%, particularly among children with severe intellectual disability, highlighting the difficulty in NREM sleep substaging in some of these patients.

In a study of 10 patients with LGS undergoing evaluation with implanted electrodes in the centromedian (CM) thalamus, patients exhibited longer wakefulness and shorter stage II sleep compared with healthy controls. Among patients with LGS, those experiencing seizures had significantly more REM periods with shorter latency than patients without seizures [19]. Another study by the same group, involving five patients with LGS, employed all-night EEG recordings using thalamic depth electrodes alongside concurrent scalp EEG to characterize both normal and abnormal stage II slow-wave sleep (SWS II) patterns [20]. A total of 1439 SWS II events (1233 normal and 206 abnormal) were analyzed using visual and statistical temporo-spatial correlation methods [20]. Most SWS II activities were concurrently observed in both scalp and CM recordings. Normal spindles, typically generated through CM–cortical interactions, were larger when synchronized and modulated other thalamo-cortical events [20]. In contrast, abnormal spindles originated from widespread cortical and CM regions, disrupting physiological rhythms. Vertex waves localized to parietal scalp regions, and overall findings implicated thalamo-cortical circuits in the generation of both normal and abnormal SWS II activities [20].

### 3.3. Sleep Microstructure and Epileptic Activity

Sleep microarchitecture has been examined in limited studies, most notably by Eisensehr et al., who analyzed the CAP, a marker of NREM instability [13]. Patients with LGS exhibited a markedly elevated CAP rate (68% vs. 33% in controls), and generalized polyspike bursts were significantly more frequent during CAP phases, particularly phase A [13]. The frequency of generalized polyspike bursts during NREM sleep correlated with both the number of A phases containing polyspikes and their mean polyspike count [13]. In adults, Sforza et al. confirmed that interictal epileptiform discharges (IEDs) were sleep stage–dependent, peaking during NREM stage 2 and slow-wave sleep, and lowest in REM [17]. The IED rate was highest during the first three hours of the night, suggesting a circadian or homeostatic influence on epileptic activity. Another study also reported more frequent interictal and ictal discharges during NREM sleep [15]. A study using thalamic depth electrodes with concurrent scalp EEG demonstrated that seizure occurrence and the duration of the late seizure component were markedly increased during stage II sleep and decreased during REM sleep. Interictal spike–wave discharges were significantly more prominent during NREM sleep and attenuated during REM sleep compared with wakefulness [19].

### 3.4. Respiratory Abnormalities and Sleep-Disordered Breathing

Sleep-disordered breathing (SDB) in LGS has received limited attention but is increasingly recognized as an important comorbidity. SDB in LGS encompasses both apneas and hypopneas, which are disruptions in airflow during sleep.

In a cohort of children with DEEs, including 14 with LGS (n = 23), patients exhibited significantly worse respiratory parameters—AHI/hour, oxygen desaturation index/hour, mean peripheral oxygen saturation (SpO_2_), and SpO_2_ nadir (all *p* < 0.001)—as well as higher periodic limb movements (PLMs%, *p* < 0.001) compared with 40 healthy controls [12]. In another study of adults with DEEs, including six with LGS, full polysomnography with respiratory monitoring revealed that over half of participants had moderate-to-severe obstructive sleep apnea (OSA; AHI ≥ 15), and nearly one-third had severe OSA (AHI ≥ 30) [18]. Tonic seizures were frequently associated with central apneas, sometimes representing their only clinical manifestation. Contributing factors included antiseizure medication–related weight gain, benzodiazepine use, and structural craniofacial abnormalities.

### 3.5. Circadian and Hormonal Rhythmicity

One study investigated circadian organization in 16 institutionalized individuals with LGS aged 8–45 years by measuring salivary melatonin, cortisol, and axillary temperature every two hours over 26 h [16]. Of the 9 subjects with normal sleep–wake patterns (group 1), 2 showed abnormalities in one or more rhythms, whereas 6 of the 7 subjects with disordered sleep (group 2) had rhythm disruptions. All three rhythms were disrupted in 2 subjects from group 2, who were also the only ones with abnormal cortisol rhythms, highlighting a higher prevalence and severity of circadian dysregulation in those with sleep disorders.

### 3.6. Ambulatory and Interventional Studies

Recent work has explored more accessible sleep assessment methods. A 2025 pilot feasibility study demonstrated that home-based wearable devices, such as the Apple Watch (consumer-grade device that tracks sleep, heart rate, and activity via photoplethysmography and accelerometry), and WatchPAT (a wrist-worn home sleep apnea testing device indicated in individuals 12 years and older) that estimates sleep stages and respiratory indices using peripheral arterial tone, heart rate, and oxygen saturation) were acceptable and practical for children with LGS, enabling multiweek monitoring outside the hospital setting [14]. The study reported an average monitoring duration of approximately 16.9 days and demonstrated that the use of wearable sleep monitoring devices was feasible in LGS. Families generally favored wearable devices over traditional modalities. Among various assessment methods, parents rated questionnaires as the most convenient (mean score 4.1 ± 0.9), followed by the Apple Watch (3.7 ± 1.6), whereas WatchPAT and polysomnography were rated as least convenient (2.4 ± 2.1 and 2.4 ± 2.0, respectively). The Apple Watch was the most preferred tool overall (1.6 ± 1.0), highlighting its practicality and user comfort in this population. The WatchPAT was moderately preferred (3.3 ± 1.4), ranking above polysomnography. In contrast, polysomnography remained the least preferred method (4.1 ± 1.4). Comparison of apnea–hypopnea index (AHI) and peripheral respiratory disturbance index (pRDI; a metric used in home sleep testing devices like WatchPAT) values showed concordance between two of the three sleep apnea tests, with one yielding discrepant results.

A registered but unpublished randomized, double-blind, crossover trial (NCT01370486) aimed to evaluate the effect of melatonin on sleep architecture and epileptiform discharges in LGS. The protocol specified PSG assessments before and after treatment, with primary outcomes including ≥50% reduction in nocturnal discharges and ≥15% increase in slow-wave sleep. However, the trial was subsequently withdrawn, and no published results are currently available.

## 4. Discussion

This scoping review identified a consistent pattern across small and heterogeneous studies: individuals with LGS exhibit profound alterations in sleep architecture, including reduced REM sleep, abnormal NREM composition, increased NREM instability characterized by elevated CAP rates, frequent nocturnal interictal and ictal discharges concentrated in NREM sleep, circadian rhythm disturbances affecting hormones and temperature regulation, and a notable but underrecognized burden of sleep-disordered breathing, particularly in adults. These findings are consistent with well-established, syndrome-independent mechanisms seen in other forms of epilepsy, but they appear more pronounced in LGS [21,22,23].

Although one study reported similar total sleep time and wake after sleep onset between individuals with LGS and healthy controls, real-world data suggest otherwise [13]. A large caregiver survey revealed substantial sleep curtailment in LGS, with 58% of patients sleeping less than seven hours per night and 18% sleeping less than five hours [4]. These findings mirror broader observations in DRE, where total sleep time and sleep efficiency are consistently reduced compared with controls [24,25,26,27]. In another study of 31 children with DEEs, including eight with LGS, total sleep time was decreased, the proportion of NREM stage 1 sleep was increased, and total REM sleep was significantly reduced compared with controls [28]. Beyond frequent nocturnal seizures, arousals in LGS may also result from sleep-disordered breathing, hypoventilation (related to hypotonia, scoliosis, or poor airway clearance), behavioral factors such as co-sleeping—which may occur in up to half of patients—and medication effects, including certain anti-seizure medications (ASMs) such as lamotrigine [4,29].

Alterations in NREM microstructure appear particularly striking. One study demonstrated markedly elevated CAP rates in LGS, with generalized polyspike bursts concentrated in CAP phase A—indicating that NREM instability provides an especially favorable substrate for epileptiform activity. High CAP and increased A1 indices have also been observed in children with epilepsy and intellectual disability [30]. Patients with DRE had increased CAP rates than self-limited epilepsy [26]. Importantly, ASMs may be able to decrease CAP rates and increase phase B duration [31]. CAP has been implicated in sleep-related cognitive processing, and early evidence links specific CAP components to learning and memory functions, underscoring the need to study CAP–cognition relationships in LGS [32].

Regarding sleep-disordered breathing, pediatric cohorts DEEs—including LGS—did not consistently demonstrate an excess of respiratory events, though mean and nadir oxygen saturation values were significantly lower in affected children [12,28]. By contrast, adult studies revealed elevated rates of both obstructive and central sleep apnea, suggesting progressive vulnerability over time, possibly influenced by medication burden, weight gain, craniofacial changes, or chronic respiratory effects of antiseizure therapies [18,33]. Systematic reviews have reported a wide prevalence range (9–65%) of sleep-disordered breathing (SDB) in children with epilepsy [34]. Evaluation of SDB is critical in LGS, as intermittent hypoxia and sleep fragmentation can trigger inflammation, autonomic dysregulation, endothelial dysfunction, and oxidative stress—all of which contribute to adverse cognitive and behavioral outcomes as well as poor cardiovascular and metabolic consequences [35,36]. Despite these risks, polysomnography remains logistically difficult and often conducted in artificial environments; home apnea monitoring has shown higher feasibility and acceptability among LGS families [14]. Identifying and managing SDB is clinically meaningful: continuous positive airway pressure (CPAP) and surgical treatments such as tonsillectomy and adenoidectomy have been shown to improve seizure control in other epilepsy populations [37,38]. The interplay between tonic seizures, brief central apneas, and autonomic dysregulation in LGS remains poorly defined, though central and complex apneas have been described in other childhood epilepsies [39].

In this scoping review, one multimodal feasibility study was included that combined home-based monitoring with polysomnography. Additionally, another study (not included in this review) also used Beacon’s FDA-cleared Dreem 3S™ EEG headband for home-based monitoring and successfully assessed sleep disturbances in both caregivers of children with LGS and the LGS participants themselves [40]. Caregivers showed significant sleep fragmentation, with Wake After Sleep Onset (WASO) of 37.79 min vs. 24.70 min in controls (*p* < 0.05) and shorter sleep onset latency (14.62 min vs. 23.43 min). LGS participants exhibited fewer sleep cycles, increased wakefulness during sleep, and a marked reduction in REM sleep (56.77 min vs. 104.34 min in controls, *p* < 0.05), reflecting both the syndrome’s detrimental impact on sleep and the practicality of at-home sleep assessment.

Although the effects of various treatments—including ASMs, dietary interventions, neuromodulation, and epilepsy surgery—on sleep have been studied in general epilepsy populations, data specific to LGS remain limited. This scoping review did not identify any treatment-focused studies, except for a single planned trial on melatonin, which was subsequently withdrawn [41]. However, patients with LGS are typically on polytherapy, combining ASMs with other treatments, and the effects of many of these combinations on sleep require further investigation [42,43,44]. Valproate appears to exert minimal sleep disruption in healthy adults but has variable effects in epilepsy, including reports of increased total sleep time and daytime naps [41]. Clobazam may improve sleep continuity by reducing stage N1 and wake after sleep onset while increasing N2, though it decreases REM sleep [41]. Lamotrigine has been associated with reduced slow-wave sleep and increased N2 and REM proportions [41]. Cannabidiol (CBD) may have favorable effects on sleep and is the most extensively studied intervention in the context of LGS, with one study involving 35 children (including 4 with LGS) reporting improvements in both sleep architecture and overall sleep quality [45,46]. Neuromodulation therapies, including vagus nerve stimulation (VNS) and deep brain stimulation (DBS), show mixed effects on sleep [47,48,49]. VNS has been associated with improved alertness but may exacerbate sleep-disordered breathing, whereas anterior nucleus (ANT) DBS elicits voltage-dependent electroclinical arousal responses [50,51,52]. Centromedian nucleus DBS, in contrast, appears to have little impact on sleep architecture [53]. The ketogenic diet (KD) may also modulate sleep architecture. In a cohort of 18 children (five with LGS), polysomnography at baseline and after KD initiation revealed a decrease in total and nocturnal sleep time but preservation of slow-wave sleep and a significant increase in REM sleep at both three and twelve months [54]. These REM improvements correlated positively with better quality of life scores [54].

Despite the predominance of nocturnal seizures in LGS, circadian biology remains largely understudied. One study, however, reported higher rates of abnormal temperature, melatonin, and cortisol rhythms in LGS, alongside disrupted day–night sleep cycles [16]. Circadian rhythms, governed by CLOCK genes and other transcriptional regulators, orchestrate sleep–wake cycles, hormone release, and autonomic activity; their expression is reduced in epileptogenic tissue, although LGS-specific data are lacking [55]. Clinically, circadian-informed ASM dosing may enhance both seizure control and sleep quality. For example, differential evening dosing of clobazam in children with nocturnal seizures led to a median seizure reduction of 75%, compared to 50% with conventional dosing [56]. Other relevant approaches may include timed light therapy, strategically scheduled melatonin, and behavioral interventions aimed at stabilizing sleep–wake patterns.

Although epileptic activity in LGS involves large-scale brain networks, its impact on sleep networks remains poorly understood. Limited evidence suggests that diffuse thalamocortical dysrhythmia and network hyperexcitability both influence and are influenced by sleep architecture in LGS [19,20,57,58]. Elevated CAP reflects unstable NREM that facilitates generalized epileptiform discharges, while REM deficiency removes a natural suppressor of seizures [13]. Together, these mechanisms heighten nocturnal epileptic burden, fragment restorative sleep, and exacerbate cognitive and behavioral impairments—mirroring sleep-mediated morbidity observed in other epileptic encephalopathies. Aberrant sleep oscillations, including reduced spindles, K-complexes, and vertex waves, further disrupt memory consolidation and cortical inhibition, emphasizing the intertwined nature of epileptiform activity, sleep disturbance, and cognitive dysfunction in LGS.

### 4.1. Limitations

This scoping review has several limitations. The primary studies were small, single-center investigations, all of which exhibited significant selection and other biases, including convenience sampling, often unmatched controls, lack of blinding in scoring, single-night assessments, and inadequate adjustment for age, etiology, or medications. Cohorts are heterogeneous with respect to age, etiology, medication regimens, and recording environments (inpatient versus ambulatory). Key factors affecting sleep and respiration—such as medication use, neuromodulation status, and comorbidities like obesity or craniofacial anomalies—are inconsistently reported. Although LGS diagnoses were consistently reported in the primary studies, they may not fully align with the current International League Against Epilepsy (ILAE) 2022 criteria due to evolving definitions [1]. The ILAE 2022 criteria are more specific than earlier definitions, focusing on multiple seizure types, characteristic EEG patterns, and cognitive impairment [59]. Some patients previously diagnosed with LGS may not meet current criteria, while others may now be classified under broader DEE categories. The impact of these changes on the interpretation of older study findings is uncertain. Many studies emphasize electrophysiologic outcomes (e.g., interictal discharge frequency, CAP indices) rather than patient-centered measures such as daytime functioning, cognition, or quality of life. Additionally, inpatient polysomnography may not reflect habitual sleep, and seizure-related apneic events shorter than 10 s are often unrecognized by standard sleep-disordered breathing scoring, despite their potential clinical relevance. Evidence regarding the relationship between tonic seizures and central sleep disturbances in LGS is very limited, with only one study reporting an association between frequent tonic seizures and central apneas, highlighting a need for further investigation. Finally, parasomnias have not been systematically studied or clearly characterized in patients with LGS, representing a knowledge gap and a potential challenge in differentiating nocturnal seizures from parasomnia-like behaviors.

### 4.2. Implications for Clinical Practice

Clinicians should maintain a high index of suspicion for sleep disturbances in LGS. (Table 2) Screening for sleep-disordered breathing using questionnaires and, when feasible, polysomnography is appropriate—particularly for adults and children with risk factors such as obesity, craniofacial abnormalities, chronic benzodiazepine use, or VNS therapy. Sleep assessment during routine EEG or overnight video-EEG can also be valuable, as careful visual analysis of the sleep portions may provide important insights. Medication choices and dosing schedules should take into account effects on REM sleep and respiratory drive. When SDB is identified, standard interventions such as CPAP, weight management, or ENT evaluation may improve not only sleep but also seizure control and daytime functioning, although direct evidence in LGS remains limited.

### 4.3. Future Research Directions

Future studies should pursue large, multicenter, prospective polysomnographic cohorts using standardized sleep scoring, full EEG montages, and harmonized reporting of ASMs, neuromodulation, body mass index (BMI), and etiology, encompassing both pediatric and adult patients to define age-related trends [60]. Ambulatory and longitudinal sleep monitoring—combining validated wearables with sleep diaries—could capture habitual sleep, seizure–sleep interactions, and long-term outcomes. Quantitative EEG and CAP analyses should be integrated with seizure timing to determine whether pharmacologic or behavioral CAP modulation reduces nocturnal epileptiform activity. Interventional trials assessing whether SDB treatment (CPAP, positional therapy, weight loss) or circadian-targeted interventions (melatonin, light therapy) improve seizures, cognition, and quality of life are urgently needed. Mechanistic studies using intracranial EEG or neuroimaging should dissect thalamocortical contributions to CAP and generalized discharges, potentially identifying novel therapeutic targets (e.g., REM/NREM modulation or orexinergic signaling). Future studies should investigate how specific genetic variants associated with LGS may contribute to distinct sleep phenotypes, which could inform personalized management strategies.

Emerging work also underscores the cognitive relevance of sleep stages: slow-wave sleep supports hippocampal–neocortical consolidation of declarative memories, while REM sleep promotes emotional memory integration and synaptic plasticity [61,62]. Disruptions in either stage correlate with cognitive decline across neurologic disorders. Future studies in LGS should test whether targeted neurostimulation—such as closed-loop slow-oscillation stimulation, thalamic or hippocampal DBS, or responsive neurostimulation (RNS) with phase-locked delivery—can enhance circadian changes in aperiodic activity, slow wave sleep, or REM architecture and improve cognitive resilience in LGS [63].

## 5. Conclusions

Although limited, the available literature paints a coherent picture: sleep in LGS is profoundly disrupted at both macrostructural and microstructural levels, characterized by NREM instability, REM reduction, and clinically relevant respiratory comorbidities in adults. These disturbances likely exacerbate seizure burden and cognitive–behavioral morbidity but may be modifiable. Future priorities include larger, methodologically rigorous longitudinal studies coupling sleep physiology with patient-centered outcomes and targeted interventional trials to determine whether improving sleep can yield measurable benefits in seizure control and quality of life for individuals with LGS.

## Figures and Tables

**Table 1:** Summary of Studies Examining Sleep in Lennox–Gastaut Syndrome (LGS).

Citation	Country/Design	Population (n, Age)	Sleep Measure(s)/Objective	Key Findings	Quality Rating (JBI)
Amir N et al. [11]	Israel/Retrospective	11 LGS (4.5–17 yrs) vs. 6 epilepsy controls vs. 12 healthy	Serial polysomnography	REM absent or markedly reduced in 5/11; mean REM % 11.2 vs. 17.8 (epilepsy) and 20.3 (controls).	Moderate–High
Carotenuto M et al. [12]	Italy (2 centers)/Unclear	23 children with refractory epileptic encephalopathies (14 LGS, mean age 8.7 ± 1.4 yrs) and 40 healthy controls	Overnight full polysomnography comparing children with EEs vs. controls	Children with EEs had reduced TIB, TST, and sleep percentage; increased REM latency, stage shifts, and awakenings; worse respiratory parameters and more PLMs.	Moderate
Clinical Trial NCT01370486	Switzerland/Registered trial	LGS (n not reported)	PSG pre/post melatonin vs. placebo; interictal discharge quantification	Hypothesized melatonin ↓ nocturnal discharges and tonic seizures, ↑ slow-wave sleep; trial withdrawn before completion.	Not feasible
Eisensehr I et al. [13]	Germany/Prospective	10 LGS (17–24 yrs; 6 M) vs. 10 controls	Polysomnography with cyclic alternating pattern (CAP) analysis	↓ Stage 2 and REM sleep; ↑ Stage 3 sleep. CAP rate 68% vs. 33% in controls. Polyspike bursts higher during CAP phase A.	Moderate
Gupta R et al. [14]	USA/Prospective	15 children (mean 12.2 yrs)	Polysomnography and Home sleep monitoring (Apple Watch, sleep logs)	Feasibility study: wearable devices preferred; comparable apnea indices across devices; high parental acceptability.	Not feasible
Horita H et al. [15]	Japan/Retrospective	9 children (2–14 yrs) with LGS	Polysomnography	↓ REM % and REM density; sleep spindles absent in 6/10; NREM substaging not possible.	Moderate–High
Laakso ML et al. [16]	Finland/Prospective	16 LGS (8–45 yrs); 9 with normal and 7 with disordered sleep–wake rhythms	26 h sampling of salivary melatonin, cortisol, and temperature	Abnormal circadian rhythms in most with disordered sleep; multiple parameters disrupted.	Moderate
Sforza E et al. [17]	Switzerland/Retrospective	13 adults (mean 30.6 ± 2.4 yrs; 71% M)	Overnight video-PSG (≥7 h)	Interictal discharges highest in Stage 2 (123/h) and SWS (106/h), lowest in REM (26/h); most frequent in first 3 h.	Moderate
Sivathamboo S et al. [18]	Australia/Prospective	13 adults with DEEs (6 LGS, 2 LGS-like; 20–50 yrs)	PSG + video-EEG with respiratory measures (AHI, SpO_2_)	53.8% had moderate–severe OSA; 30.8% severe. Frequent tonic seizures linked with central apnea and fragmented sleep.	Moderate
Velasco AL et al., 1995 [19]	Mexico/Retrospective	10 LGS (4 adults; mean 23.2 and 6 children; mean 5.3); historical controls	All-night EEG with bilateral centromedian thalamic depth and scalp electrodes	Patients with LGS showed significantly longer wakefulness and shorter stage II sleep than normals; and that LGS patients with seizures showed significantly larger numbers and shorter latency of REM periods than those without seizures. The occurrence of seizures and the duration of the late component significantly increased during stage II sleep and decreased during REM sleep. Interictal spike-wave discharges were significantly larger during NREM and smaller during REM than during wakefulness.	Moderate–High
Velasco AL et al., 2002 [20]	Mexico/Retrospective	5 LGS (4–26 yrs; mean 11.6)	All-night EEG with bilateral centromedian thalamic depth and scalp electrodes	Normal and abnormal SWS II activities characterized; abnormal spindles from widespread cortical/CM regions disrupted normal rhythms.	Moderate–High

↓ = decrease; ↑ = increase.

**Table 2:** Practical Recommendations for Sleep Care in Lennox–Gastaut Syndrome.

Recommendation	Details/Notes
Routine Screening	Screen for sleep disturbances and sleep-disordered breathing (SDB) in all patients with LGS, particularly adults, those with obesity, craniofacial anomalies, or vagus nerve stimulation (VNS).
Polysomnography (PSG)	Order PSG when there is snoring, witnessed apneas, excessive daytime sleepiness, or unexplained seizure exacerbation.
EEG-PSG Interpretation	Interpret EEG and PSG jointly, as NREM instability and CAP activation often coincide with epileptic discharges and may influence nocturnal seizure frequency.
Medication Review	Assess antiseizure medications (ASMs) for sedative or REM-suppressing effects (e.g., benzodiazepines, barbiturates) and adjust if sleep quality is compromised. ASMs may also be responsible for insomnia.
SDB Management	Treat identified SDB with CPAP or other standard approaches; although evidence in LGS is limited, improvements in seizure control and alertness are plausible.
Sleep Hygiene & Caregiver Counseling	Encourage regular sleep–wake schedules, consistent bedtime routines, and minimize nocturnal seizure triggers such as sleep deprivation or illness.
Specialist Collaboration	Collaborate with sleep specialists for complex cases and management of comorbid circadian rhythm disturbances.
Research & Monitoring	Encourage enrollment in studies using wearable or home-based monitoring to refine sleep–epilepsy biomarkers in LGS.

Abbreviations: LGS—Lennox–Gastaut syndrome; SDB—sleep-disordered breathing; VNS—vagus nerve stimulation; PSG—polysomnography; EEG—electroencephalography; NREM—non-rapid eye movement; CAP—cyclic alternating pattern; ASMs—antiseizure medications; CPAP—continuous positive airway pressure.

## Data Availability

Not applicable.
